# *In planta* Female Flower Agroinfiltration Alters the Cannabinoid Composition in Industrial Hemp (*Cannabis sativa* L.)

**DOI:** 10.3389/fpls.2022.921970

**Published:** 2022-07-21

**Authors:** Michihito Deguchi, Seema Dhir, Shobha Potlakayala, Sarwan Dhir, Wayne R. Curtis, Sairam Rudrabhatla

**Affiliations:** ^1^The Central Pennsylvania Research and Teaching Laboratory for Biofuels, Penn State Harrisburg, Middletown, PA, United States; ^2^Biology Department, Fort Valley State University, Fort Valley, GA, United States; ^3^Family Sciences and Technology, College of Agriculture, Fort Valley State University, Fort Valley, GA, United States; ^4^Department of Chemical Engineering, The Pennsylvania State University, University Park, PA, United States

**Keywords:** agroinfiltration, cannabidiol, *Cannabis sativa* L., metabolic engineering, tetrahydrocannabinol

## Abstract

Industrial hemp is a diploid (2n = 20), dioecious plant, and an essential source of various phytochemical productions. More than 540 phytochemicals have been described, some of which proved helpful in the remedial treatment of human diseases. Therefore, further study of hemp phytochemicals in medicine is highly anticipated. Previously, we developed the vacuum agroinfiltration method, which allows the transient gene expression in hemp tissues including female flowers, where cannabinoids are produced and accumulated. In this study, we attempted to alter the composition of total CBD and THC. The RT-PCR and sanger sequence identified eleven copies of the CBDAS gene, two copies of the THCAS gene, and one CBCAS gene. Binary vectors were constructed to overexpress the CBDAS gene and silence the THCAS gene via RNA interference. The Transcript level of the CBDAS gene was increased by more than 10 times than the plants used as a control, which led to a 54% higher total CBD content. The silencing of the THCAS gene led to downregulation of the THCAS gene, with an 80% reduction in transcript levels, and total THC content was reduced to 43% compared with mock plant. These results suggest that hemp vacuum infiltration is highly effective for metabolic engineering of cannabinoids in hemp.

## Introduction

Hemp (*Cannabis sativa* L.) is an important multipurpose crop with a rich source of fiber, essential fatty acids, easily digestible proteins (albumin and edestin), and enhanced levels of the amino acid arginine. This crop's pharmaceutical compounds have been studied more recently since they show potent bioactivities on human diseases (Schluttenhofer and Yuan, 2017).

**Figure 1:**
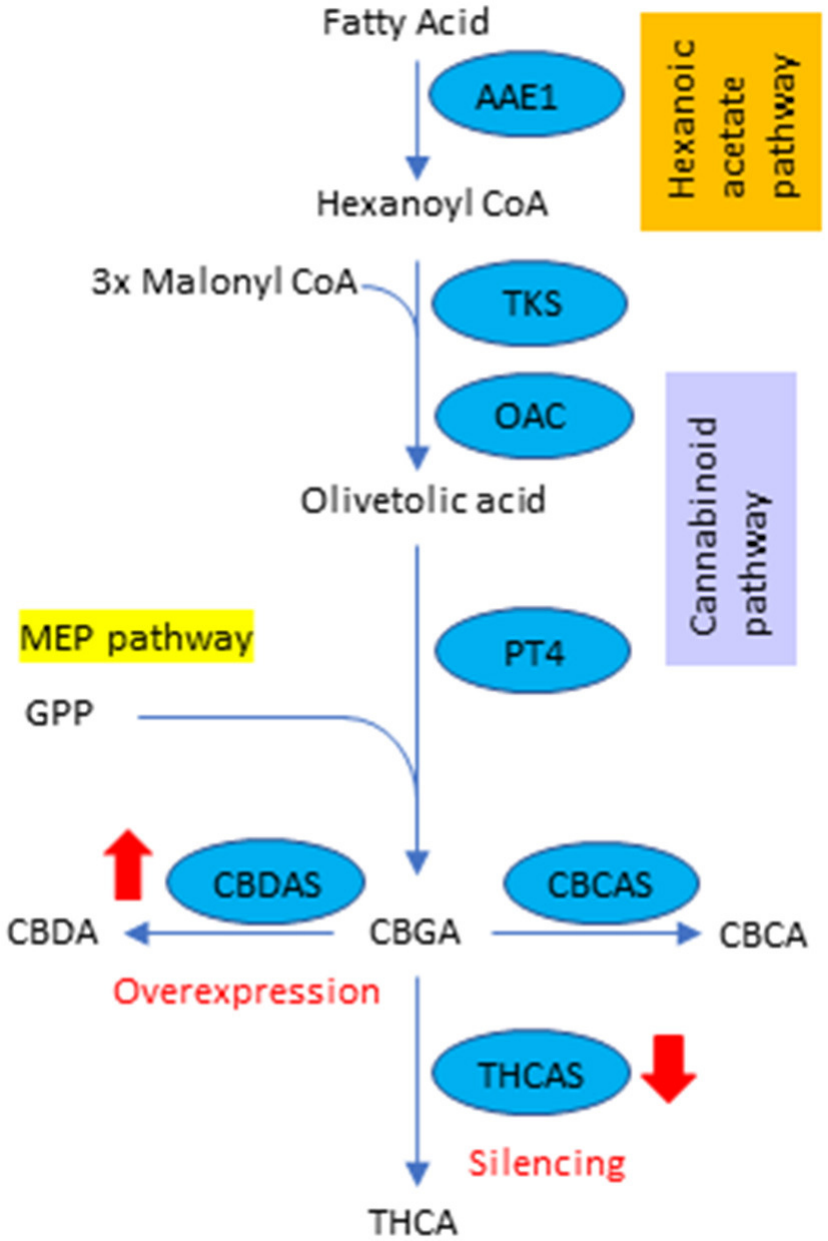
The schematic pathway of phytocannabinoid synthesis and target genes in hemp.

Cannabinoids are the secondary metabolites in hemp and are produced in capitate stalked glandular trichomes, located predominantly on female flowers (Livingston et al., 2021). So far, 120 cannabinoids have been identified, among which there is mostly a growing interest in two: Cannabidiol (CBD) and tetrahydrocannabinol (THC), due to their high pharmacological properties (Izzo et al., 2009). The cannabinoid pathway is summarized in Figure 1. The pathway is initiated by synthesizing olivetolic acid, which originates from the primary metabolite precursor, hexanoyl-CoA, by tetraketide synthase (type III polyketide synthase) and olivetolic acid cyclase, in the cytosol (Gülck and Moller, 2020). Olivetolic acid is transported to plastids and converted into cannabigerolic acid (CBGA) by CBGA synthase using geranyl diphosphate (GPP) from the plastidial 2-C-methyl-D-erythritol-4-phosphate (MEP) pathway. Afterward, CBGA is transferred to apoplastic space to synthesize cannabidiolic acid (CBDA), tetrahydrocannabinol acid (THCA), and Cannabichromene acid (CBCA) by CBDA synthase (CBDAS), THCA synthase (THCAS) and CBCA synthase (CBCAS), respectively. Some part of CBDA, THCA and CBCA are decarboxylated to CBD, THC and CBC by non- enzymatic reactions, respectively. Other significant cannabinoids such as cannabinol (CBN) and tetrahydrocannabivarin (THCV) are also present at certain concentrations in female flowers (Tahir et al., 2021).

CBD is the primary cannabinoid in hemp and has shown potential therapeutic agents for several central nervous system diseases such as epilepsy, neurodegenerative diseases, schizophrenia, multiple sclerosis, affective disorders, and the central modulation of feeding behavior (Andre et al., 2016). The first CBD-based product, epidiolex, was recently approved by the U.S. Food and Drug Administration as an anticonvulsant drug (Pauli et al., 2020). On the other hand, hemp also produces low amounts of THC, which is 0.3% on the dry weight basis or less. The THC has therapeutic benefits because it causes relief from nausea and possesses analgesic, neuroprotective, anticancer, anti-inflammatory, and anti-diabetic properties (Hussain et al., 2021). However, THC has been associated with several side effects, such as sensations of euphoria, paranoia and anxiety, cognitive and cholinergic deficits, and immunosuppression (Andre et al., 2016).

Since there is an increased demand for high CBD content and concerns about the presence of a psychoactive agent, we aimed to increase CBD and remove the THC from our hemp cultivar “CRS1” by agroinfiltration-mediated metabolic engineering. Stable transformation is the first choice for plant genetic engineering to add valued traits. However, hemp stable transformation is still a challenge because of its low regeneration efficiency. To combat this, we developed a transient gene expression system in a previous report by vacuum agroinfiltration as an alternative to stable transformation (Deguchi et al., 2020a). Herein, we applied the vacuum agroinfiltration protocol to in planta female flowers of hemp to alter the expression levels of cannabinoid synthase genes and change the cannabinoid contents into desirable profiles.

## Materials and Methods

### Plant Material, Greenhouse Conditions, Generation of Clones, Growth, and Care

The hemp strain, “CRS1,” was grown following the approved guidelines for industrial hemp provided by the Pennsylvania Department of Agriculture - Bureau of Plant Industry under the regulated permits IH-16-P-2017 and IH-17-P-2017.

Hemp clones were achieved by collecting a 3-inch segment containing two axillary buds and coating the 45-degree cut with Clonex Rooting gel (Hydrodynamics International, Inc. Lansing, MI). The explant was placed in Root Riot plugs (Hydrodynamics International, Inc. Lansing, MI) and maintained under propagation domes for 2 weeks. Subsequently, they were transferred to four-inch pots containing high porosity soil, HP Mycorrhizae from Pro-Mix (Rivière-du-Loop, Québec, Canada). The clones grew to 15 cm by week four and flowered by week nine. Vegetative cuttings were collected from the same female mother plant to produce genetically identical clones of similar size. The greenhouse conditions were maintained at 25°C with a 14-hour light photoperiod during vegetative growth stage and 12-hours light photoperiod during pre-flowering and flowering stages at 25–40μEm-2s-1.

The humidity for rooting clones was maintained at 65% and decreased gradually to 45% once the clones started to flower. Lost Coast Plant Therapy (Plant Protector, Inc. Loleta, CA) was applied to the clones biweekly at a dilution of 30 mL per 4 liters to control pests.

### Cloning, Sequencing, and Phylogenetic Analysis of Cannabinoid Synthase Genes

Total RNA was extracted from 100 mg of each plant sample using the SpectrumTM Plant Total RNA kit (Sigma Aldrich, St. Louis, MO, USA). RNA concentration and absorbance ratios (A260/280 and A260/230) were measured using a NanoVue Plus spectrophotometer (General Electric Healthcare Limited, UK) to measure the quantity and quality of the total RNA. After treatment with DNase I (TaKaRa Bio, Dalian, China) to remove genomic DNA contamination, 2 μg of total RNA was used to synthesize cDNA using the high-capacity cDNA reverse transcription kit (Applied Biosystems, Foster City, CA) according to the manufacturer's protocol. The cannabinoid synthase genes were amplified with Platinum Taq DNA Polymerase High Fidelity (Invitrogen, Carlsbad, CA, USA) using mix primers. Forward mix primer sequences were composed of 5'-ATATTTTTCTTTTTCTC-3' and 5'-ATATTTTTCTTTCTCTC-3', and reverse mix primer includes 5'-CTTTGTTCGTTTCTAAA-3' and 5'-CTTTGCTCGTTTCTAAA-3'. The objective size of bands was isolated from agarose gel electrophoresis, purified and cloned into pJET1.2, blunt cloning vector using Clone JET PCR Cloning kit (Fermentas, Burlington, Canada). Those plasmids were sent to Macrogen (Seoul, Korea) for a sanger sequence of insert genes. Phylogenetic analysis was conducted based on the unique CBDAS, THCAS, and CBCAS gene sequences with the Maximum Parsimony method of the MEGA7 software package (Tamura et al., 2011). This software was also used for the alignment of cannabinoid synthase genes.

### Construction of Binary Vectors and Agroinfiltration

**Figure 2:**
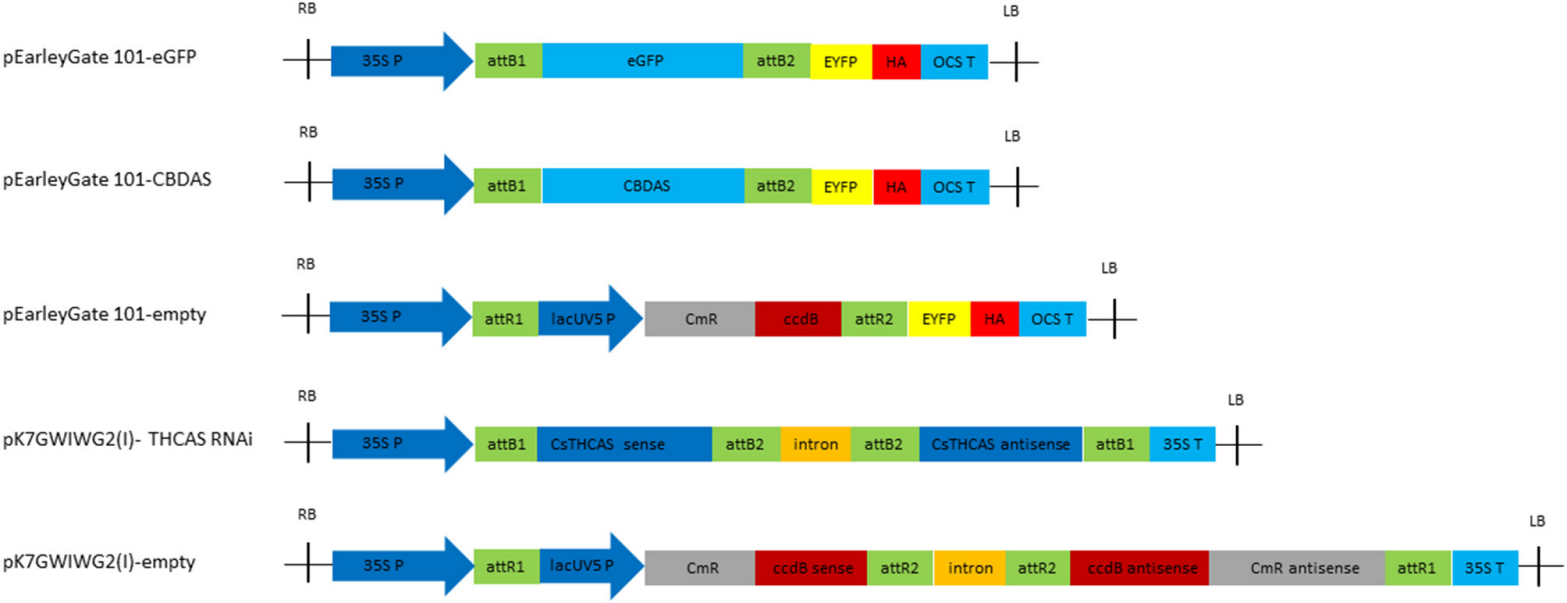
Schematic diagram of the metabolic engineering constructs in this study.

pEarleyGate 101 vector (Invitrogen, Carlsbad, CA, USA) harboring the eGFP gene and CBDAS gene (AB292682) under the control of a Cauliflower mosaic virus (CaMV) 35S promoter and OCS terminator was used for GFP fluorescence assays and CBDAS overexpression, respectively. An empty pEarleyGate 101 vector was used as a negative control. To silence the THCAS gene, the hairpin RNA-expressing RNAi construct was prepared by inserting sense and antisense partial THCAS fragments into the pK7GWIWG2 gateway vector (Invitrogen). A 209 bp partial sequence (THCAS 992nt−1200nt) was amplified in the sense orientation using cloning primers which include recombination sequences, attB1 and attB2, in forward and reverse primers, respectively, to insert into the entry vector, Gateway pDONR/Zeo/Zeo (Termo Fisher Scientific, Waltham, MA, USA). Targeted gene fragment in the entry clone was then transferred to the binary silencing vector, pK7GWIWG2(I), to form the hairpin RNA, which will be targeted by dicer to synthesize mature small RNA. An empty pK7GWIWG2(I) was used as a negative control. All binary vector cassettes were summarized in Figure 2.

The construct was evaluated by sequencing and subsequently transformed into Agrobacterium tumefaciens GV3101 cells. Agrobacterium culture was resuspended in agroinfiltration media containing 10 mM MES, 1x MS, 2% glucose, 0.015% Silwett L-77 (Lehle seeds, Round Rock, USA), 0.05% Pluronic F-68 (Gibco, NY, USA), 5 mM L-Ascorbic acid (Sigma-Aldrich, St. Louis, MO, USA) and 200 μM acetosyringone at a pH 5.6.

Agroinfiltration was performed as previously described (Deguchi et al., 2020a). Hemp female plants produced from the same mother plant were used, and 10–14 days old female flowers in the fourth branch from the top were agroinfiltrated via vacuum infiltration. Three independent female plants were agroinfiltrated per each binary vector. Hemp female plants were placed in a vacuum chamber and female flowers were dipped in *Agrobacterium* solution. The vacuum pump was turned on to decrease the pressure, and the agroinfiltration time was calculated after the vacuum reached 80 mbar. Successful infiltration required bubbles to be flowing up from the hemp female flower. After 10 min at low pressure, the release valve was opened slowly to allow entrance of *Agrobacterium* into the interstitial spaces of the plant tissues. The flower tissues were then washed with distilled water to avoid overgrowth of Agrobacterium, transferred onto a moist filter paper in a petri dish and placed in a growth room at 21°C. Four days after agroinfiltration, female flowers were harvested and stored in the−80°C freezer until further analysis.

### Image Collection, Quantification of Binary Vector, Analysis of Gene Expression and Cannabinoid Content

Image collection and analysis of GFP fluorescence were conducted as previously described (Deguchi et al., 2020a). Four days after vacuum agroinfiltration, the hemp female flowers were subject to GFP fluorescence observation using a Nikon SMZ1500 microscope with Imaging Software NIS Elements F Package (Nikon) equipped with a DS-Ril1 camera (Nikon), an Intensilight C-HGFI Precentered Fiber Illuminator (Nikon) and a GFP2 filter set (Ex. 480 ± 40 nm; Em. 510 nm LP). Agroinfiltration rate was measured by quantifying binary vectors in hemp flowers via qPCR. According to the manufacturer's protocol, DNA was extracted from agroinfiltrated female flowers (100 mg) using the DNeasy Plant Mini Kit (Qiagen, Valencia, CA, USA). qPCR was performed with 5 μL of SYBR Select Master Mix (Applied Biosystems, Waltham, MA, USA) in a 10 μL total reaction mixture containing 400 nM of primers and 1 μL of DNA (100 ng/μL). The kanamycin-resistant gene was amplified to quantify the pEarleyGate 101 vector with the following primers (Fw: 5'- ATGGCTAAAATGAGAATATC−3', Rv: 5'- CTAAAACAATTCATCCAGTA−3'), and spectinomycin resistant gene was amplified to quantify pK7GWIWG2(l) vector with following primers (Fw: 5'-ATGGGGGAAGCGGTGATCGC-3', Rv: 5'- TTATTTGCCGACTACCTTGG-3'). To make a calibration curve, Ct values at 5 different concentration series of binary vectors (50 ng/μL, 5 ng/μL, 500 pg/μL, 50 pg/μL, 5 pg/μL, and 500 fg/μL) were measured using Bio-Rad CFX96 system (Bio-Rad, Hercules, CA, USA) under the following reaction conditions: Initial denaturation at 95°C for 10 min, 40 cycles of 95°C for 10 s, and 60°C for 1 min. The copy number of plasmids at each concentration was calculated using the molecular weight and plasmid concentration.

The gene expression of two cannabinoid pathway genes, CBDAS and THCAS was measured using qRT-PCR (Deguchi et al., 2021). Since the CBDAS and THCAS genes have more than 90% homology in their sequences, both sequences were aligned, and a variable region was used to design qRT-PCR primers to avoid off-target effects between them. The primer sequences of CBDAS are Fw: 5'-GATCCGCTGGGCAGAACGGT-3' and Rv: 5'- ATGAGGGAATGGAATTGCTG-3', and the primer sequences of THCAS are Fw: 5'- GATCAGCTGGGAAGAAGACG-3' and Rv: 5'- ATGAGGGAATGGAATTGCTG-3'. The elongation factor 1-α gene was used as a reference gene (Fw: 5'- GCCCTGTCTTTGAGAGCAAC-3', Rv: 5'-CAATCCACTGCTCAATGTGG-3'). Relative gene expression levels of CBDAS and THCAS were calculated using the 2^−ΔΔCt^ method (Deguchi et al., 2021). Statistical analysis was performed using a 1-way ANOVA with Tukey's multiple comparison test (α = 0.05). Three independent biological replications were conducted.

Cannabinoid contents were measured by Analytica 360 (Yakima, WA, USA). Collected flower samples were first dried in an convection oven for 6–24 h at 90°C. The 400 mg of dry tissue was extracted with 50% methanol at 60°C for 10 min. The extracts were filtered using a Spin-X column (Corning Inc., Corning, NY, USA). HPLC analysis was performed using a Waters 2695 system coupled to a Waters 3100 single quadrupole mass detector and a 996 photodiode array detector. A reverse-phase Zorbax C-18 column 4.6 mm × 100 mm, 3.5 μ m was eluted by using a mixture (85:15) of methanol and ultrapure water as the mobile phase in an isocratic system with a flow rate of 1.0 ml/min. The standard solutions were prepared to construct calibration curves for the quantification of following cannabinoids: Δ9-THCA, Δ9-THC, Δ8-THC, CBDA, CBD, CBGA, CBG, CBC (Sigma-Aldrich, St. Louis, MO, USA). Peak identification of cannabinoids was performed by comparing the retention times of the samples with those of the standard solutions.

## Results

### Phylogenetic Analysis and Sequence Alignment of Cannabinoid Synthase Genes

**Figure 3:**
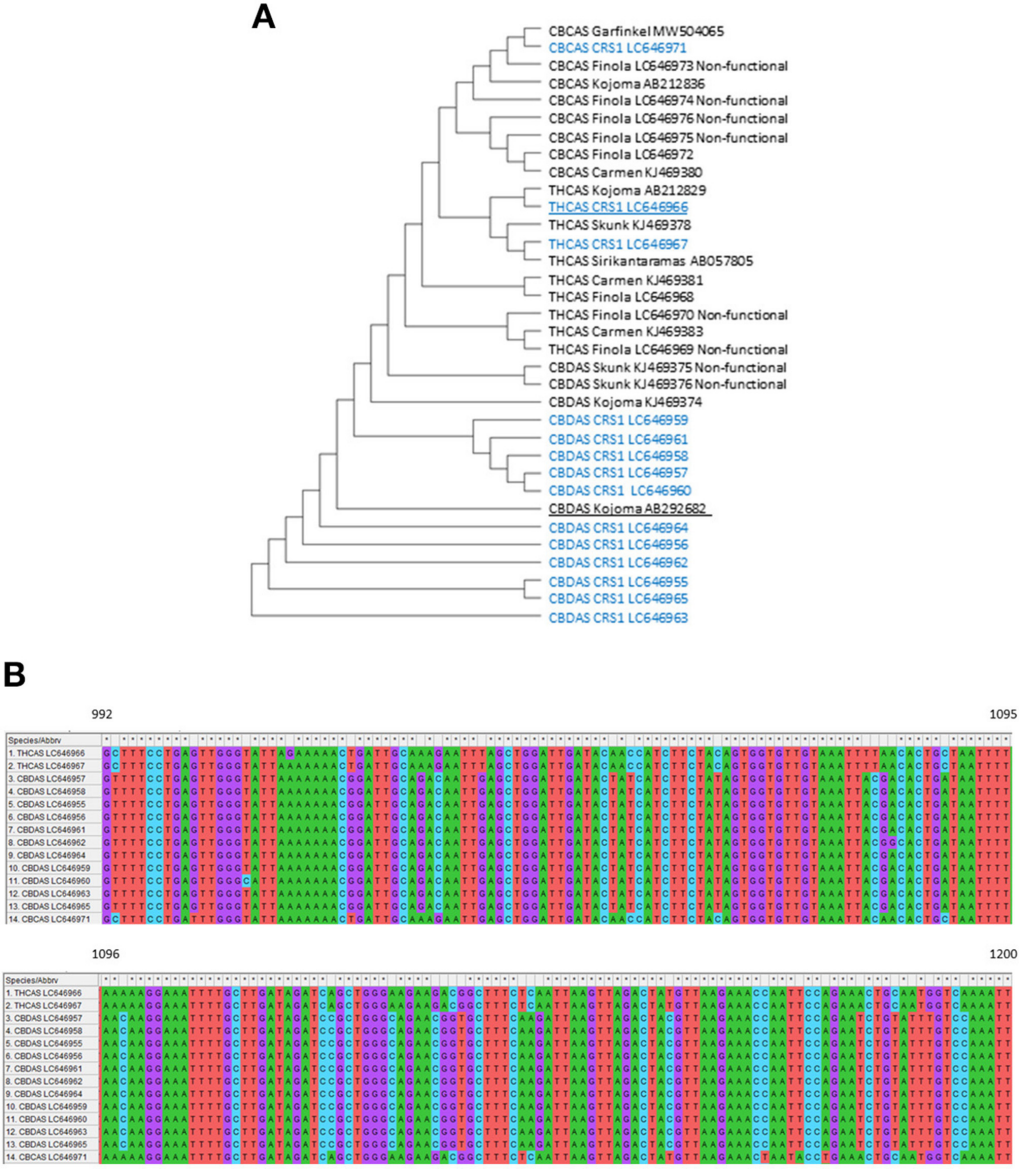
**(A)** Construct/test maximum parsimony tree of the CBDAS, THCAS, and CBCAS gene sequences inferred from a 1452 bp alignment of 14 unique sequences obtained from RT-PCR in “CRS1” (No. of Bootstrap Replications were 2000). The blue letter represents expressed sequences in “CRS1.” Underlined copies were used to build binary vectors. **(B)** Sequence alignment of CBDAS, THCAS, and CBCAS from 992nt-1200nt. All sequences were obtained from RT-PCR in “CRS1”.

The cannabis genome is highly heterozygous and contains large amounts of repetitive elements. Cannabinoid synthases represented by CBDAS, THCAS, and CBCAS exist in large copy numbers due to gene duplication and divergence (Hurgobin et al., 2021), which requires detailed sequence analysis to select target sequences for the genetic engineering of hemp. We first retrieved functional and non-functional forms of CBDAS, THCAS, and CBCAS from the NCBI database (https://www.ncbi.nlm.nih.gov/) (Figure 3A). Next, we designed mix primers at conserved sequence regions adjacent to start and stop codons and carried out RT-PCR to isolate expressed cannabinoid synthase genes from “CRS1.” The 1456-nt partial fragments of CBDAS, THCAS and CBCAS were isolated and sequenced. We obtained 11 unique CBDAS copies, two unique THCAS copies, and one unique CBCAS copy (Figure 3A). They code 485 amino acids and did not include any stop codon. Notably, non-functional sequences present in the hemp genome were not isolated from any of THCAS, CBDAS and CBCAS, implying that truncated cannabinoid synthase genes are not expressed or expressed at an exceptionally low level. All isolated CBDAS homologs have high homology (>99%) and were identified to contain several single nucleotide polymorphisms (SNPs) leading to different protein translations. CBDAS Kojoma AB292682 which was introduced into yeast for the synthesis of CBDA, was used for the construction of the overexpression vector below (Luo et al., 2019). Two THCAS copies, LC646966 and LC646967, shared high homology (>99%) and possessed five and two SNPs with THCAS Sirikantaramas AB057805, which was used to synthesize THCA in N. tobacco (Sirikantaramas et al., 2005). To find appropriate THCAS fragments for hairpin construction, all isolated CBDAS, THCAS, and CBCAS gene sequences were aligned in MEGA to find variable regions (Figure 3B). The 209 nucleotides between 992nd to 1200th from the starting codon possess lower homology to CBDAS (83–85%) than other regions. Hence, we used this region to clone a THCAS hairpin cassette.

### Female Hemp Flower Vacuum Agroinfiltration

**Figure 4:**
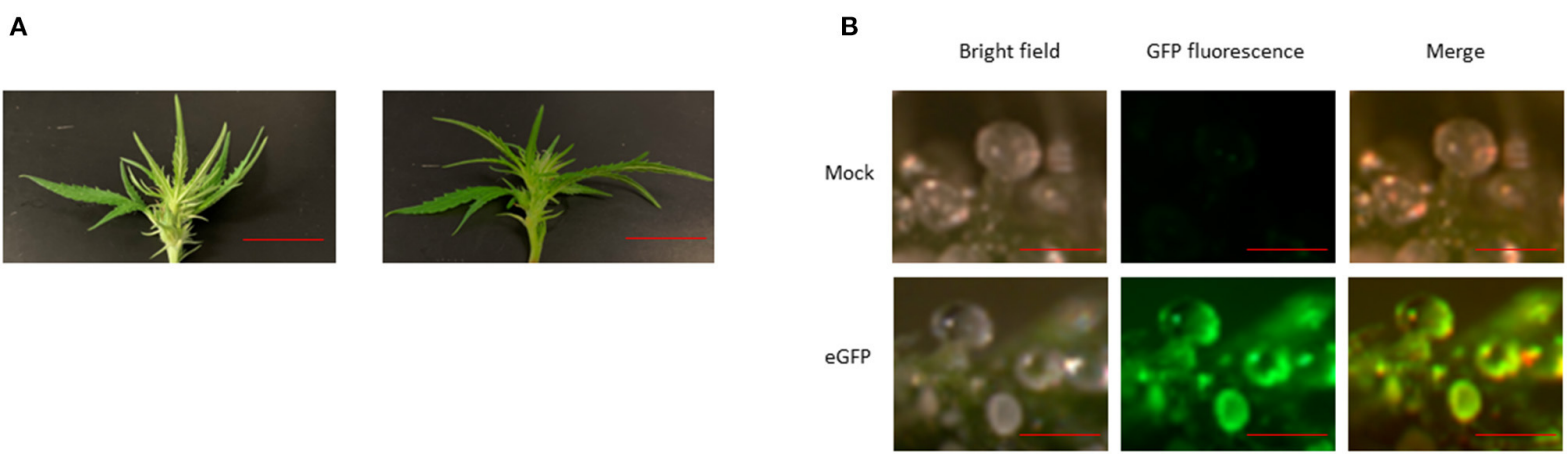
**(A)** Female hemp flower before (left) and after (right) agroinfiltration. Bar 1.5 cm. **(B)** Transient GFP expression in the female flower. Bar 1.2 mm.

For overexpression of CBDAS, we used full-length cDNA of CBDAS Kojoma AB292682 and cloned into pEarleyGate 101 vector that was driven by CaMV35S promoter and OCS terminator (pEarleyGate 101-CBDAS, Figure 2). To silence THCAS, we built pK7GWIWGE(l)-CsTHCAS RNAi, which consists of inverted repeats of THCAS coding sequence separated by an intron. pEarleyGate 101-eGFP was used as a reporter gene (Deguchi et al., 2020a). All binary vectors were transformed into Agrobacterium GV3101 and subjected to vacuum agroinfiltration in mature female flower. Wetting of the flower tissue was observed after agroinfiltration, which indicates successful infiltration (Figure 4A). Next, GFP cassette, pEarleyGate 101-eGFP was infiltrated, and GFP fluorescence was observed in whole stalked glandular trichomes including the trichome head (Figure 4B).

### Absolute Quantification of Binary Vectors in Agroinfiltrated Female Flowers

**Figure 5:**
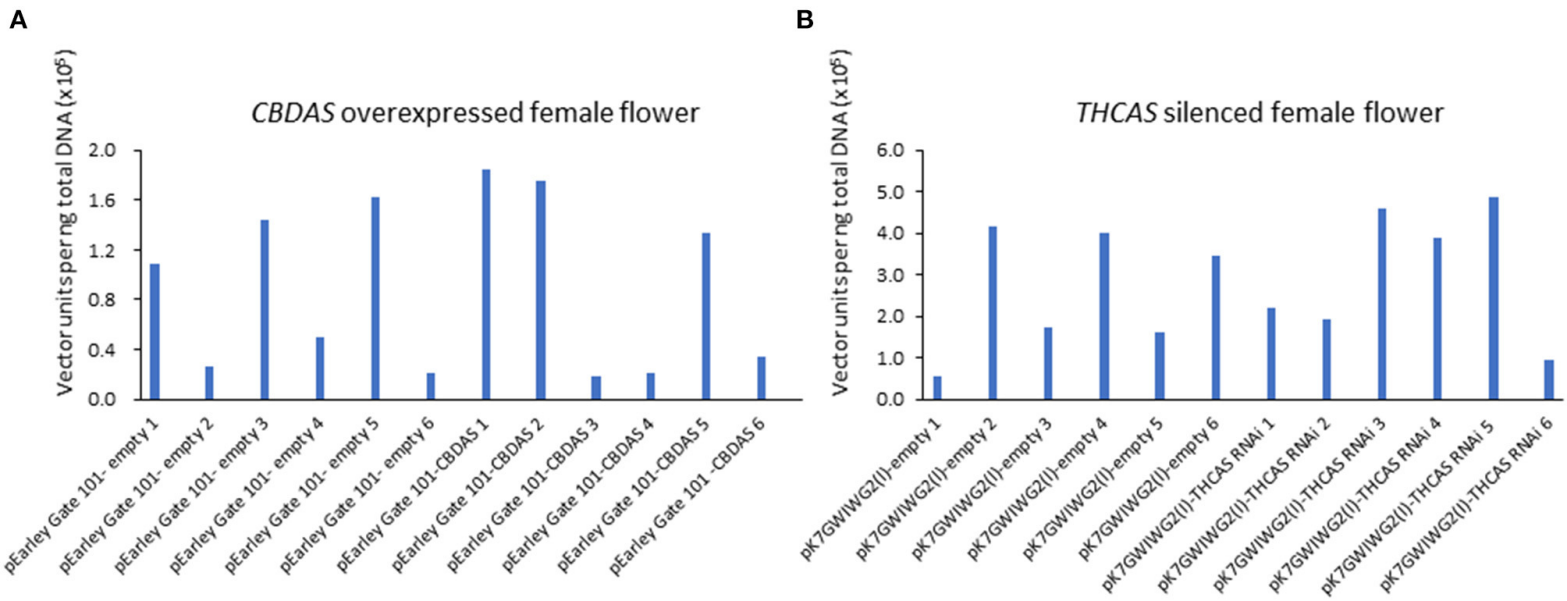
Absolute quantitation of vector units in agroinfiltrated hemp flowers in *CBDAS* overexpressed female flowers **(A)** and THCAS silenced female flowers **(B)**. The vector accumulation was determined by qPCR using a calibration curve.

Afterward, we evaluated the agroinfiltration rate by quantifying the introduced vector units via qPCR (Figures 5A,B). In the treatment for CBDAS overexpression, vector unit value (VUV) was high at pEarleyGate 101-CBDAS line 1 (VUV = 1.84), 2 (VUV = 1.75), and 5 (VUV = 1.33), whereas line 3 (VUV = 0.19), 4 (VUV = 0.21) and 6 (VUV = 0.36) showed lower VUV (Figure 5A). In mock plants for CBDAS overexpression, VUV was higher at pEarleyGate-empty line 1 (VUV = 1.1), 3 (VUV = 1.4), 5 (VUV = 1.6), and lower at line 2 (VUV = 0.26), 4 (VUV = 0.50) and 6 (VUV = 0.22). For the silencing of THCAS, VUV was higher at pK7GWIWG2(l)-THCAS RNAi line 3 (VUV = 4.58), 4 (VUV = 3.89) and 5 (VUV = 4.88), although line 1 (VUV = 2.19), 2 (VUV = 1.94) and 6 (VUV = 0.93) showed lower VUV (Figure 5B). In mock plants for the silencing of THCAS, pK7GWIWG2(l)-empty lines 2 (4.17), 4 (VUV = 3.99), and 6 (VUV = 3.46) showed higher VUV, and line 1 (VUV = 0.55), 3 (VUV = 1.73) and 5 (VUV = 1.66) showed lower VUV. As a result, those 12 lines showing higher agroinfiltration rates were subjected to the following gene expression and cannabinoid content analysis.

### Gene Expression Analysis of *CBDAS* and *THCAS*

**Figure 6:**
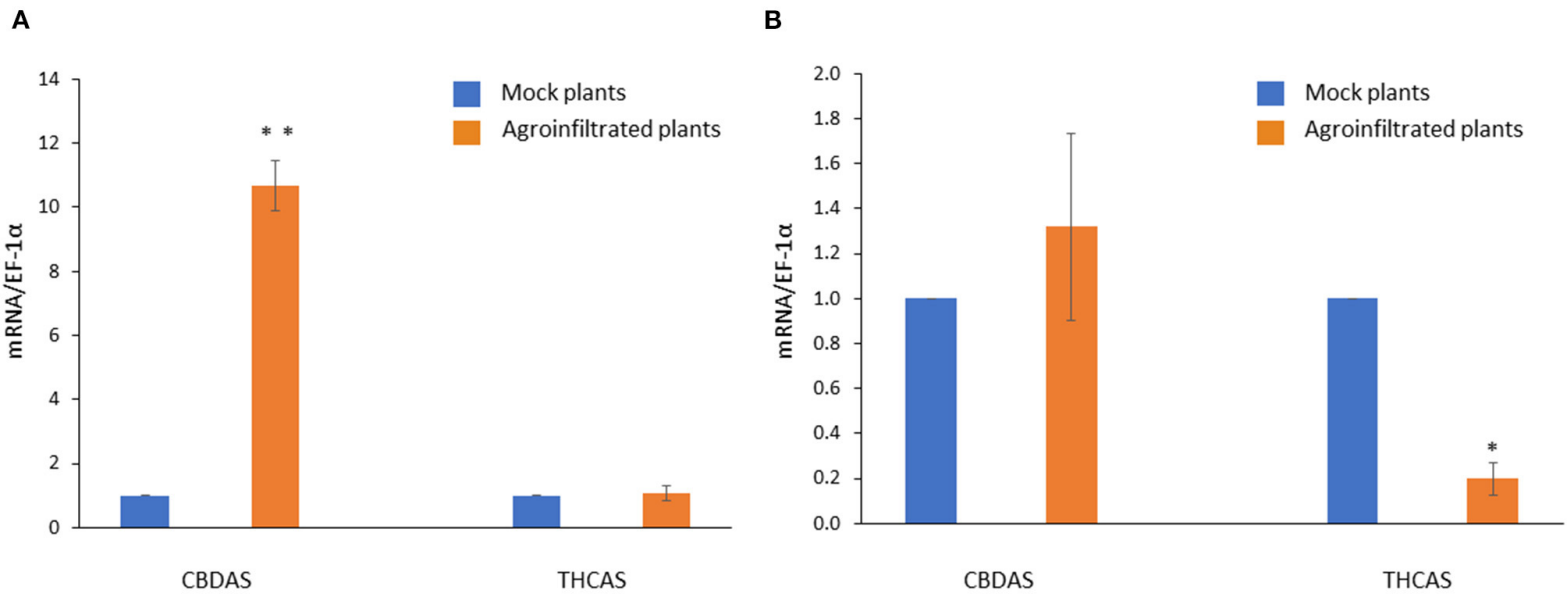
Gene expression profiles of *CBDAS* and *THCAS* in *CBDAS* overexpressed female flowers **(A)** and *THCAS* silenced female flowers **(B)**. Data is presented as mean ± S.E. * and ** indicates statistically significant differences at *P* < 0.05 and *P* < 0.01, respectively, compared with mock plants, according to the statistical analysis in paired *t*-tests.

The overexpression of CBDAS resulted in CBDAS expression being more than ten times higher than that of mock plants (Figure 6A). There was no significant difference in THCAS expression between pEarleyGate 101-CBDAS infiltrated plants, and pEarleyGate 101-empty infiltrated plants. On the other hand, pK7GWIWG2(l)-THCAS RNAi caused downregulation of THCAS expression with an 80% reduction (Figure 6B). There was no significant difference in CBDAS expression between pK7GWIWG2(l)-THCAS RNAi infiltrated plants and pK7GWIWG2(l)-empty infiltrated plants, demonstrating that there was no off-target effect of hairpin THCAS RNA on the CBDAS expression.

### Cannabinoid Contents in Agroinfiltrated Female Flowers

In hemp female flowers, Δ9-THCA, CBDA, CBD, CBGA were detected, and Δ9-THC, Δ8-THC, CBG and CBC were below the limit of quantification.

**Figure 7:**
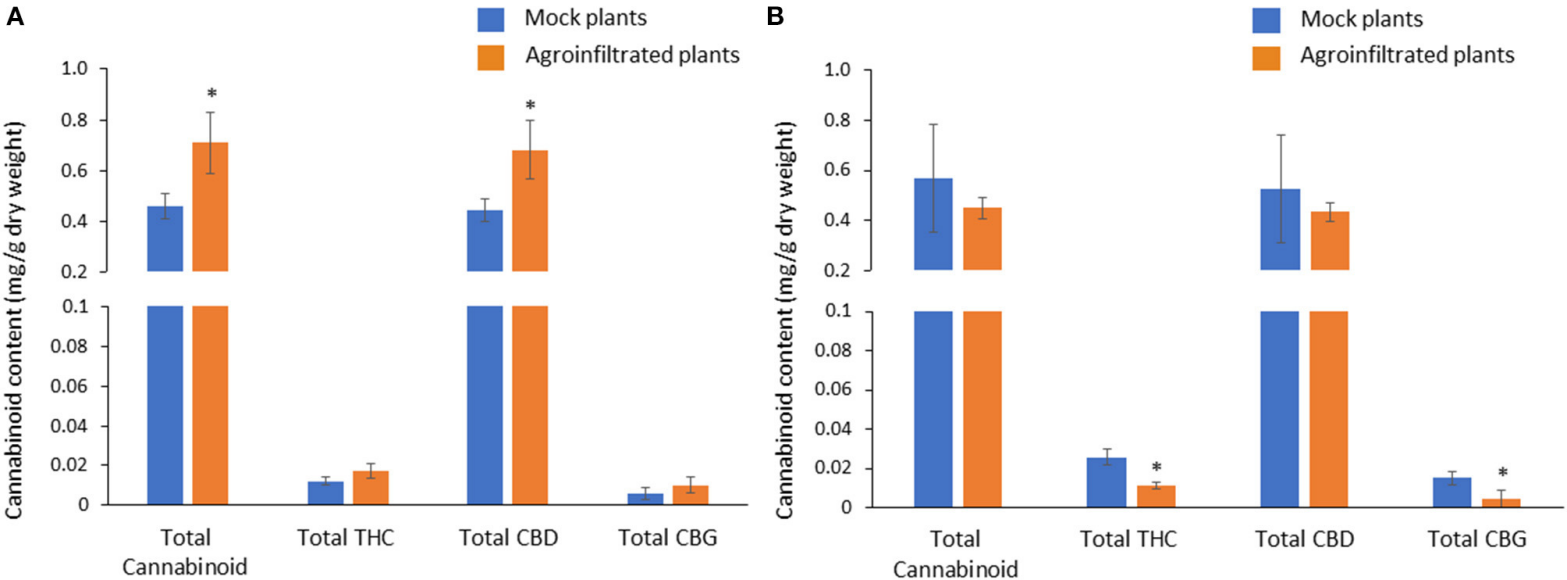
Comparison of cannabinoid contents between mock and agroinfiltrated plants in *CBDAS* overexpressed female flowers **(A)** and *THCAS* silenced female flowers **(B)**. The cannabinoid contents were quantified with HPLC. Data is presented as mean ± S.E. *Indicates statistically significant differences at *P* < 0.05 compared with mock plants, according to the statistical analysis in paired *t*-tests.

Overexpression of CBDAS gene resulted in 54% higher total cannabinoid and total CBD (Figure 7A). There was no effect of CBDAS overexpression on total THC and CBG. Likewise, silencing of the THCAS gene brought a significant reduction of total THC, which is equivalent to 57% of total THC in mock plants. In contrast, it did not affect total cannabinoid or total CBD (Figure 7B). Notably, CBG was also decreased with a 71% reduction compared with the mock plants.

## Discussion

There are several successful reports for Cannabis stable transformation (Galán-Ávila et al., 2021; Hesami et al., 2021; Zhang et al., 2021), however, they still show very low efficiency and genotype dependence. More experimental research is required, including optimizing various parameters such as age and type of explant, type and concentration of plant growth regulators, macro and micronutrients, and vitamins (Hesami et al., 2021). As an alternative method to stable transformation, we previously reported that vacuum agroinfiltration is helpful for both overexpression and silencing in excised hemp tissues/organs. Here, applying this agroinfiltration system to the in planta female flowers, the expression of cannabinoid synthase genes was altered, which led to the modification of cannabinoid composition. This work is the first successful metabolic engineering of cannabinoids in Cannabis sativa, demonstrating that our agroinfiltration system enabled gene expression changes at high levels to alter the composition of phytochemicals.

The copy number variations of cannabinoid synthases have been reported in the Cannabis genome (Laverty et al., 2019; McKernan et al., 2020) and are likely to have resulted from genome duplication and tandem rearrangement of long terminal repeat retrotransposons (Grassa et al., 2021; Hurgobin et al., 2021). CBDAS exists in significantly larger copy numbers than THCAS and CBCAS (Weiblen et al., 2015; Vergara et al., 2017). This is consistent with our results that eleven copies of CBDAS were isolated from cDNA libraries synthesized from female flowers whereas only two copies of THCAS and one copy of CBCAS were isolated (Figure 3A). Additionally, we found the functional copies of CBDAS to be highly similar (>99% nucleotide identity) among accessions. This might be because intensive breeding practices have been performed to select these desirable cannabinoid synthase loci, which led to them being less polymorphic. Notably, there are many non-functional copies of cannabinoid synthase genes in the Cannabis genome. Nevertheless, no non-functional copy was identified in RT-PCR and sequencing. These results suggest that one or a few copies are predominantly responsible for synthesizing CBDA, THCA, and CBCA, as suggested previously (Hurgobin et al., 2021).

In planta flower agroinfiltration has been performed for the study of the flower petal color in ornamental plants (Shang et al., 2007; Hussein et al., 2013; Ratanasut et al., 2014; Fresquet-Corrales et al., 2017; Zeinipour et al., 2018). Unlike previous studies on in planta flower agroinfiltration, we performed agroinfiltration on female hemp flowers because it produces a high amount of cannabinoids in stalked glandular trichomes: a cellular metabolite factory (Tanney et al., 2021), which is abundant on the calyces and bracts in the female flower. The cannabinoids, such as CBDA and THCA, have high toxicity to the plant cell and thus induce death via apoptosis (Andre et al., 2016). To avoid the cannabinoid's toxicity, they are synthesized within secretory disk cells that line the base of the glandular trichome head. Afterward, they are transferred and stored in the secretory cavity, which was developed between secretory disk cells and the cuticle. When the pEarleyGate 101-eGFP vector was expressed via agroinfiltration, GFP fluorescence was detected in both the secretory disk cell and the secretory cavity, suggesting that our agroinfiltration system will help alter the expression of genes responsible for cannabinoid synthesis, transport and storage in stalked glandular trichome (Figure 4B).

To evaluate the efficiency of agroinfiltration in each plant, we measured the vector concentration in female flowers using the absolute quantification method. One indicator of successful agroinfiltration is wetting in infiltrated tissues (King et al., 2015). We confirmed that entire female flowers became wet because of the penetration of Agrobacterium solution into the tissues (Figure 4A). Nevertheless, infiltration efficiency varied on the individual plant, and the difference between the highest and lowest efficiency of infiltration was as much as 10 times in the same treatment. This clarified that more efforts are necessary to improve the *in planta* female flower agroinfiltration in hemp; If this method would be applied to the high-throughput study of gene function like commonly used model plants for transient expression such as Arabidopsis, Nicotiana benthamiana and Nicotiana tobacco. In previous report, we vigorously studied the effects of surfactant, antioxidant, vacuum time, Agrobacterium, and hemp strain on the efficiency of agroinfiltration (Deguchi et al., 2020a). To further improve this transient gene expression protocol, keeping plants in the dark after infiltration, a suitable developmental stage and temperature for plant growth would need to be optimized (Burman et al., 2020; Zhang et al., 2020).

To date, agroinfiltration has been proven to be effective for metabolic engineering of representative secondary metabolites such as terpenoid (Reed and Osbourn, 2018; Mani et al., 2021), alkaloid (Li et al., 2019; Mora-Vasquez et al., 2022) carotenoid (Rodriguez-Concepcion and Daros, 2022), anthocyanin (Fresquet-Corrales et al., 2017) as well as minor secondary metabolites (van Herpen et al., 2010; Pan et al., 2021), most of which were achieved in N. benthamiana. This plant species shares typical cellular compartmentalization, cofactor, and coenzymes with other plants, enabling it to introduce new synthetic pathways without extensively optimizing the system. Moreover, N. benthamiana produces recombinant proteins at high levels and is amenable to Agrobacterium; thus, it does not cause necrosis. Because of these reasons, N. benthamiana usually becomes the first choice of a platform for heterologous phytochemical production. Gülck et al. (2020) attempted heterologous production of CBDA by overexpression of five cannabinoid pathway genes– AAE1, TKS, OAC, PT, CBDAS– via agroinfiltration in N. benthamiana. Although high expression of these exogenous genes was detected, none of the cannabinoids were synthesized. This is likely due to the different localization of these enzymes; more specifically, olivetolic acid is synthesized by AAE, TKS and OAC at cytosol. Whereas, CBGA synthesis from olivetolic acid and GPP occurred by PT at plastid, and CBDA is synthesized from CBGA and geranyl diphosphate by CBDAS at apoplast in Cannabis sativa (Hurgobin et al., 2021).

On the other hand, we previously optimized and established hemp agroinfiltration methods (Deguchi et al., 2020a). In this work, this protocol was applied to in planta agroinfiltration on female flowers, leading to a drastic increase in CBDAS expression (Figure 6A) and 54% higher CBDA content than control plants (Figure 7A). The effect of enhanced CBDAS expression on total CBD content was not as high as expected despite CBDAS expression being highly increased (Figures 5A, 6A). These results suggest that a couple of agroinfiltrations from the earlier flowering stage might be effective for the continuous high CBDAS expression to achieve a higher accumulation of total CBD. Based on metabolite analysis, the precursors for cannabinoid synthesis are not likely to be abundant (Figure 7A; Gagne et al., 2012), thus, further supply of precursors from primary metabolite will also be essential. AAE1 is a first step enzyme in the cannabinoid pathway and is responsible for the conversion of fatty acids to hexanoyl-CoA. Stout et al. (2012) revealed that the concentration of hexanoyl-CoA paralleled the accumulation of the CBDA, indicating the AAE1 gene will be a rate-limiting step in CBDA synthesis. Hence, simultaneous overexpression of both CBDAS and AAE1 will lead to a higher accumulation of CBDA than CBDAS overexpression alone.

Moreover, in planta agroinfiltration to female flowers was highly effective to silence the THCAS gene via RNA interference (Figure 7B). The copy number of the THCAS gene is likely to be one or two, based on genome sequences which were consistent with the fact that we obtained only two different copies of THCAS (Matchett-Oates et al., 2021a). We used a 209 bp fragment (THCAS 992nt-1200nt) to make a hairpin construct which seemed to result in a non-off target effect on the CBDAS (Figure 5B). There is approximately only 85% homology within this region between THCAS copies and CBDAS copies. Interestingly, Matchett-Oates et al. (2021b) reported successful downregulation of CBDAS, THCAS, and CBCAS via RNAi using agroinfiltration. They used a 603 bp fragment of the THCAS gene for siRNA generation. Their RNAi construct did not only show a significant reduction of THCAS with 60% downregulation but also 70% and 40% downregulation of CBDAS and CBCAS, respectively, because of off-target effects. These results imply that smaller RNAi constructed using variable sequence regions might be more effective to avoid or reduce off-target effects on the silencing of cannabinoid synthase genes. Additionally, total CBG content was also decreased in *THCAS* silenced female flower, however, there was no increase in total CBD or total CBC. These results implies that the synthesis of other cannabinoids, for example, CBN, THCV and CBDVA might have been activated by silencing of *THCAS*.

In this work, we successfully increased total CBD content and decreased THC content by the vacuum agroinfiltration method in female hemp flowers. Previous reports proposed that CBDAS is a rate-determining step for CBDA synthesis (Taura et al., 2007; Husain et al., 2019), and our result was consistent with this hypothesis. Although there is high copy number variation on CBDAS gene in the hemp genome sequence, 11 CBDAS copies isolated from female flowers had more than 99% homology, and truncated copies were not detected. Likewise, only two individual THCAS copies (>99% homology) were isolated from female flowers. These results highly encourage us to challenge knocking out the CBDAS gene and the THCAS gene to boost the production of CBGA or eliminate THCA from female flowers, which is desired by the hemp industry. To perform complete and specific downregulation of cannabinoid synthase genes, genome editing technology, particularly CRISPR/Cas9, would be the best choice. This technology enables the knockout of several homologous genes via a single editing step (Jacobs et al., 2017). Recently, several successful cannabis transformations have been reported (Galán-Ávila et al., 2021; Zhang et al., 2021). A combination of CRISPR/Cas technology with hemp regeneration and stable transformation protocols with high efficiency will generate a lot of new strains with added values (Shiels et al., 2022). These hemp plants will be classified into cisgenic plants, which are easier to obtain public acceptance for commercialization (Deguchi et al., 2020b).

## Conclusion

Here, our vacuum agroinfiltration protocol proved to be very useful for gene overexpression/silencing studies to alter the composition of cannabinoids in female hemp flowers. The protocol will also expedite the function studies on genes responsible for not only secondary metabolite synthesis but also flowering time and sex determination, which are associated with important traits for hemp growth but remain to be unraveled (Petit et al., 2020).

## Data Availability Statement

The original contributions presented in the study are included in the article/supplementary material, further inquiries can be directed to the corresponding author.

## Author Contributions

MD contributed to the experimental design, prepared plant materials, analyzed the data, prepared the figures, and wrote the original manuscript of the paper. SR, SP, SeDhir, and SaDhir revised manuscript. SR was responsible for project administration, funding acquisition, resources, and supervision of this study. All authors read and approved the final version of the paper.

## Funding

We want to acknowledge support from the P.A. Options for Wellness and Penn State Harrisburg School of Science, Engineering, and Technology.

## Conflict of Interest

The authors declare that the research was conducted in the absence of any commercial or financial relationships that could be construed as a potential conflict of interest.

## Publisher's Note

All claims expressed in this article are solely those of the authors and do not necessarily represent those of their affiliated organizations, or those of the publisher, the editors and the reviewers. Any product that may be evaluated in this article, or claim that may be made by its manufacturer, is not guaranteed or endorsed by the publisher.
